# SimNav-XR: an extended reality platform for mobile robot simulation using ROS2 and Unity3D

**DOI:** 10.3389/frobt.2026.1708161

**Published:** 2026-02-18

**Authors:** Prakash Aryan, Sujala Deepak Shetty, V. Kalaichelvi, R. Karthikeyan

**Affiliations:** 1 Institute of Computer Science, University of Bern, Bern, Switzerland; 2 Department of Computer Science, Birla Institute of Technology and Science, Pilani, Dubai Campus, Dubai International Academic City, Dubai, United Arab Emirates; 3 Department of Electrical and Electronics Engineering, Birla Institute of Technology and Science, Pilani, Dubai Campus, Dubai International Academic City, Dubai, United Arab Emirates; 4 Department of Mechanical Engineering, Birla Institute of Technology and Science, Pilani, Dubai Campus, Dubai International Academic City, Dubai, United Arab Emirates

**Keywords:** autonomous navigation, extended reality, mixed reality, mobile robots, robotics simulation, ROS2, Unity3D, virtual reality

## Abstract

**Introduction:**

This paper presents SimNav-XR, an extended reality platform that integrates XR technologies with modern robotics frameworks to support mobile robot simulation and development.

**Methods:**

By connecting ROS2’s communication infrastructure with Unity3D’s rendering and XR capabilities through the ROS-TCP-Connector package, SimNav-XR provides a practical bridge between robotics middleware and game engine environments for visualization and testing. The platform implements components for physics-based robot modeling, LiDAR and IMU sensor simulation, environmental interaction dynamics, and XR interfaces supporting both Virtual Reality (VR) and Mixed Reality (MR) modes. These capabilities create interactive environments where developers can visualize and control simulated robots through immersive interfaces using the Meta Quest 3 headset with controller-based input.

**Results:**

Experimental evaluations using established platforms (Turtlebot3 and ROSbotXL) demonstrate the framework’s capabilities across virtual testing scenarios, showing successful autonomous navigation with obstacle avoidance and simultaneous localization and mapping (SLAM). The VR mode provides fully immersive virtual environments for development and testing, while the MR mode uses passthrough cameras to overlay virtual robots onto real-world surfaces via plane detection.

**Discussion:**

XR visualization techniques provide insights into robot sensor data and navigation behavior, supporting robotics development and education through accessible simulation environments.

## 1 Introduction

**FIGURE 1:**
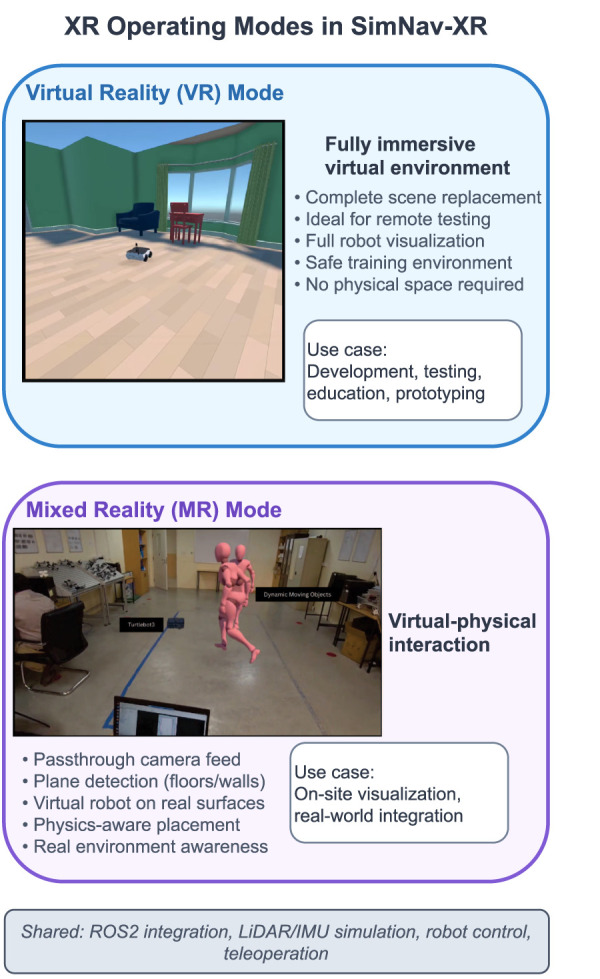
XR operating modes in SimNav-XR. Virtual Reality (VR) mode provides fully immersive virtual environments ideal for development, testing, and education. Mixed Reality (MR) mode uses passthrough camera feed and plane detection to place virtual robots on real-world surfaces, supporting on-site visualization. Both modes share the same ROS2 integration, sensor simulation, and robot control interfaces.

Extended Reality (XR) represents a technological convergence that includes Virtual Reality (VR) and Mixed Reality (MR), fundamentally transforming how humans interact with digital environments and creating new possibilities for robotics development. VR creates fully immersive digital environments that replace the physical world, while MR supports interactions between physical and virtual elements through passthrough displays and spatial awareness. Figure 1 illustrates these two operating modes as implemented in SimNav-XR. While these technologies have gained significant traction in gaming, education, and enterprise applications, their potential impact on robotics development and deployment remains underexplored. The combination of XR with modern robotics frameworks presents opportunities to improve how we design, test, and deploy robotic systems (Clark, 1996; Coronado et al., 2023). The robotics industry faces several fundamental challenges that have historically impeded rapid advancement. Traditional robot development requires extensive physical infrastructure, poses safety risks during testing, and often fails to capture the full spectrum of operational scenarios robots might encounter. The financial barriers are substantial, with hardware costs, maintenance requirements, and specialized testing facilities putting advanced robotics research out of reach for many institutions. Moreover, the increasing complexity of robotic systems demands testing methodologies that can safely explore edge cases without risking damage to expensive hardware or injury to human operators. These challenges are particularly acute in domains such as disaster response robotics, medical robotics, and space exploration, where real-world testing opportunities are limited (Murphy, 2014; Li and Yang, 2025).

Current approaches to robotics simulation typically rely on simplified physics models and limited environmental interactions, creating a “reality gap” between simulated and real-world performance (Collins et al., 2021; Li et al., 2024). Traditional simulation platforms often struggle to accurately represent complex physical interactions, sensor behaviors, and environmental dynamics. Additionally, existing simulation tools frequently lack intuitive interfaces for human interaction and control, making it difficult for developers to naturally interact with and modify robot behavior. The limitation of conventional 2D displays in representing 3D spatial relationships and robot configurations further complicates the development process (Hoffmann, 2007). The emergence of ROS2 as a robust robotics middleware framework, combined with the maturation of game engine technology exemplified by Unity3D, creates new possibilities for addressing these challenges. ROS2’s architectural improvements over its predecessor, particularly in areas of real-time performance, security, and multi-robot coordination, provide a solid foundation for developing robotics applications. Unity3D’s rendering capabilities, physics simulation, and native support for XR devices offer tools for creating immersive simulation environments. Recent advances in GPU-accelerated simulation frameworks (Mittal et al., 2025) and unified benchmarking platforms (Geng et al., 2025) have further demonstrated the potential for high-fidelity robotics simulation.

This technological convergence enables novel approaches to robotics development such as real-time interaction with simulated robots in virtual environments, transitions between different reality modes for development and testing, and intuitive interfaces for robot programming and control. Beyond the immediate technical benefits, this integration addresses aspects of robotics development that have been historically underserved. The ability to rapidly prototype and iterate robot designs in virtual environments can reduce development cycles and costs. Virtual environments can simulate scenarios that would be impractical or impossible to test physically. The integration of visualization capabilities through XR interfaces provides insights into robot operation and behavior. Developers can visualize sensor data and planning algorithms in context. The concept of digital twins has emerged as a framework for bridging physical and virtual robotic systems, enabling real-time monitoring, simulation, and optimization (Qin et al., 2026; Malik et al., 2024).

In the educational context, this framework offers possibilities for teaching robotics and control theory. Students can safely experiment with robot programming and control in virtual environments before working with physical hardware. The immersive nature of XR interfaces makes complex concepts more tangible and engaging, while the ability to visualize abstract concepts like control algorithms and sensor data helps bridge the gap between theory and practice. The system’s support for remote learning and collaboration is relevant in today’s distributed educational environment. This research introduces a framework that uses these technological advances to support mobile robot development. By combining ROS2’s robotics ecosystem with Unity3D’s game engine capabilities and XR interfaces, we facilitate robot simulation and control while maintaining compatibility with existing robotics infrastructure. The framework’s modular architecture ensures extensibility and adaptability to future advances in both robotics and XR technologies.

The key contributions of this work include: development of a unified XR framework that integrates VR and MR capabilities for robot development and testing, allowing developers to leverage the strengths of each modality while maintaining consistent interfaces and behaviors; implementation of a physics-based simulation environment that models robot dynamics, sensor behaviors, and environmental interactions using Unity3D’s physics engine; creation of intuitive human-robot interaction through XR interfaces, providing natural ways to visualize, control, and monitor robot behavior in simulated environments; and demonstration of the framework’s functionality through testing with multiple robot platforms (Turtlebot3 and ROSbotXL) in navigation tasks.

Through technical analysis and evaluation, we demonstrate the framework’s effectiveness across various robotics applications. The remainder of this paper is structured as follows: Section 2 provides a literature review of extended reality applications in robotics. Section 3 details our methodology, describing the system architecture, ROS2 integration with Unity3D, robot modeling techniques, and implementation of key components including SLAM, navigation, and sensor data processing. Section 4 presents results demonstrating the framework’s effectiveness in creating immersive virtual environments and robot visualization. Section 5 concludes with a discussion of contributions, limitations, and directions for future research.

## 2 Literature review

Extended reality (XR), an umbrella term consisting virtual reality (VR), augmented reality (AR), and mixed reality (MR), is changing the way humans interact with robots and establishing new possibilities for robotics development. XR technologies provide immersive and interactive experiences that are transforming how robots are programmed, controlled, and integrated into various industries and applications.

### 2.1 Recent advances in VR/AR technologies

Recent research has contributed to virtual and augmented reality applications for robotics development. In multi-user training environments, Braun et al. (2022) presented a prototype for immersive firefighter training on a container ship that simulates difficult-to-access environments using motion-capture systems. For feature matching in multi-view scenarios, Zhao et al. (2022) proposed DSD-MatchingNet using deformable convolution networks for robust correspondence. Collaborative interaction in VR has been advanced by Liu et al. (2024), who demonstrated benefits of sharing eye movement and gesture visualization among users. Performance optimization for VR rendering has advanced through approaches such as the neural foveated super-resolution technique by Ye et al. (2024), which uses human visual perception characteristics to reduce computational demands. Similarly, Xu T. et al. (2024) addressed rendering of complex volumetric environments through efficient binocular rendering with coupled adaptive ray marching. In gesture recognition, Karambakhsh et al. (2019) developed a convolutional neural network for augmented reality applications in medical education. These technologies complement the work of Zhao et al. (2024), who applied GAN-based techniques to image stylization. In medical robotics, Ujiie et al. (2024) developed a VR simulation system for preoperative planning of robotic-assisted thoracic surgery using head-mounted displays, while Su et al. (2024) introduced BioVRbot, a bioinspired VR toolkit for robot-assisted medical applications. High-fidelity simulation has been advanced by Embley-Riches et al. (2025), who presented Unreal Robotics Lab integrating photorealistic rendering with accurate physics simulation. These advances have influenced the design of SimNav-XR, informing our approach to visualization and simulation fidelity.

### 2.2 XR for robot programming, system engineering, and training

XR is being utilized across multiple categories in industrial robotics: robot programming, system engineering, and personnel training (Karunarathna et al., 2023; Stepanyants and Romanov, 2024). These applications span various domains (Tian et al., 2023; Luo et al., 2023) and have shown benefits for industrial automation (Devic et al., 2024; Tarng et al., 2024). Recent studies have highlighted their effectiveness in educational environments (Roman-Ibanez et al., 2018; Ujiie et al., 2024) and medical training (Bric et al., 2016). Robot Programming approaches using XR, especially AR, assist programmers by offering better understanding of the physical context. For example, AR can superimpose virtual robot trajectories onto the real-world environment, allowing programmers to visualize and validate robot motions before executing them on physical robots (Moshayedi et al., 2023; Dogangun et al., 2025). System Engineering applications of XR allow for virtual prototyping and testing of robotic systems, helping engineers identify and address potential problems early in the design process. This can reduce development time and costs compared to traditional methods. Personnel Training through XR creates immersive training environments for operators, allowing them to learn how to use and interact with robots in a safe and controlled setting. This can accelerate the learning process, improve knowledge retention, and reduce the risk of accidents during training on physical robots.

### 2.3 Using game engines for XR development in robotics

Game engines like Unity and Unreal Engine have emerged as powerful tools for creating XR applications in robotics (De La Pena Lopez et al., 2022; Collins et al., 2021). Various research has demonstrated their effectiveness for simulation environments (Roman-Ibanez et al., 2018; Coronado et al., 2023) and integration with robotic systems (Singh et al., 2024; Chaudhary et al., 2021). They offer several key capabilities for robotics development. 3D Environment Creation capabilities of game engines allow developers to create realistic 3D environments that simulate real-world scenarios. These environments can be populated with virtual robots, objects, and humans, providing an immersive experience for users. Physics Simulation features in game engines have built-in physics engines that allow for realistic simulation of robot movement, interaction with objects, and environmental conditions (Wijaya et al., 2024; Crespo et al., 2015b). This allows developers to test robot behavior and control algorithms in a variety of scenarios before deploying them on physical robots (Liu et al., 2022). XR Hardware Support in game engines supports a wide range of XR hardware, including VR headsets (Jarecki and Lee, 2024; Su et al., 2024) and AR devices (Cruz Ulloa et al., 2024; Chen et al., 2009). Additional studies have explored motion tracking systems (Crespo et al., 2015a), making it easier for developers to create XR experiences that are compatible with different devices and platforms. Scripting and Programming interfaces in game engines provide scripting and programming interfaces that allow developers to implement custom logic, control robot behavior, and integrate with external systems such as ROS. Recent comparative studies have examined the trade-offs between different game engines and traditional robotics simulators (Embley-Riches et al., 2025; Sterk-Hansen et al., 2023).

### 2.4 Connecting XR applications with robotic systems

To establish a connection between XR applications and robotic systems, communication mechanisms and software frameworks are important for supporting real-time interaction and feedback (Sobczak et al., 2022; Zofka et al., 2020). Recent research has explored various integration approaches (Manas-ilvarez et al., 2023; Suomalainen et al., 2020) and standardized protocols (Kumar et al., 2022). The Unity AR Foundation framework (Unity Technologies, 2025) provides cross-platform support for AR development, while recent work has examined ROS2 integration strategies for real-time robotic applications (Singh et al., 2024). Communication Mechanisms mentioned in the sources include TCP/IP sockets and Bluetooth as examples of communication protocols for connecting XR applications with robots (Zhang et al., 2023). TCP/IP sockets allow for reliable data transmission over networks, providing real-time control and feedback between the XR environment and the physical robot. Bluetooth can be used for short-range wireless communication between XR devices and robots. Software Frameworks like ROS represent widely used open-source frameworks for robot software development that provide tools for communication, control, and simulation. ROS allows developers to integrate XR applications with robots using packages like ROS# and Rosbridge Suite, which facilitate data exchange between Unity and ROS. Recent developments in ROS2 have introduced improved real-time capabilities and security features that benefit XR-robotics integration (Sterk-Hansen et al., 2023). The development of digital twin frameworks has further advanced these integration capabilities (Malik et al., 2024; Gonzalez-Herbon et al., 2024; Qin et al., 2026).

### 2.5 Specific examples of XR applications in robotics

The sources highlight several examples of how XR is being used in various robotics applications (Dianatfar et al., 2021; Guerra et al., 2019). Teleoperation of Legged-Manipulator Robots studies have compared MR (using Microsoft HoloLens) and VR (using HTC Vive) interfaces for teleoperating legged-manipulator robots in search and rescue scenarios. Researchers developed VR and MR interfaces with similar functionality for controlling a quadruped robot equipped with a manipulator arm. They evaluated both interfaces using metrics related to mission management, robot control, and operator performance. The results showed that both interfaces could effectively control the robot, but VR was more suitable for remote operations due to its higher autonomy, while MR was better for on-site operations because it provided a better understanding of the robot’s surroundings (Cruz Ulloa et al., 2024). VR Simulator for Robot Learning research developed a VR simulator using Unity 3D and the Oculus Rift headset to improve the learning experience for students studying robotics. The simulator featured a five-degree-of-freedom robotic manipulator and an interactive user interface that provided guidance and feedback to prevent errors. By using the simulator, students could practice programming and operating the robot in a safe and immersive virtual environment, which improved their understanding of robot kinematics and control concepts (Crespo et al., 2015b). AR-Assisted Robot Programming research explored the use of AR for simplifying and visualizing robot kinematics. They argued that visualizing complex robot movements in AR could help students grasp the concepts more effectively. One study focused on developing an AR-based system for programming industrial robots, allowing users to interact with a virtual robot overlaid on the real-world environment using hand gestures and a smartphone (Devic et al., 2024). VR-Based Training for Industrial Robots studies investigated the use of VR to teach students how to operate industrial robots remotely via the internet. The findings suggested that VR could improve their understanding of robotics. VR for Teleoperation in Hazardous Environments research developed a VR-based system for teleoperating robots in hazardous environments. The system used 3D reconstruction techniques, Google Images, and video streams to create a dynamic virtual environment that mirrored the real world. This allowed operators to control robots remotely while feeling immersed in the environment, improving situational awareness and safety (Bavelos et al., 2025; Luu et al., 2025). MR for Enhanced Collaboration in Teleoperation studies proposed a novel MR telecollaboration system that combined a robotic arm-mounted stereo camera with VR and 3D reconstruction techniques. This system enabled remote users wearing a VR headset to actively control their viewpoint by moving their heads, which manipulated the robotic arm in the local environment. This allowed for more natural and intuitive interaction during teleoperation tasks, improving collaboration between remote and local users (Hoebert et al., 2024; Zhang et al., 2023). Recent work has also explored kinesthetic teaching approaches using mixed reality interfaces (Maccii et al., 2024) and VR-based human-robot interaction for manipulation tasks (Xu W. et al., 2024).

### 2.6 Challenges and future opportunities

While XR offers benefits to robotics development, there are also challenges and opportunities for future research and development. Ergonomics and Comfort issues with current XR headsets can be uncomfortable to wear for extended periods, which limits their practicality in real-world applications. Improving the ergonomics and comfort of XR devices is important for wider adoption. Cost and Accessibility concerns arise from high costs associated with some XR devices and powerful computers needed to run complex simulations, which can limit accessibility, particularly for educational institutions and researchers with limited budgets. Developing more cost-effective XR solutions and exploring cloud-based platforms could address this challenge. Technical Limitations in tracking accuracy, latency, and field of view in XR systems can affect the fidelity of the virtual experience and the precision of robot control. Advancements in XR technology are needed to overcome these limitations. Cybersecurity concerns become important as XR systems become more integrated with critical infrastructure and industrial applications.

XR is poised to play a more significant role in robotics as the technology matures and becomes more accessible. Future research and development efforts should focus on addressing the challenges mentioned above while exploring new applications and interaction paradigms.

### 2.7 Comparative analysis of XR-robotics platforms

To situate SimNav-XR within the existing landscape of XR-enabled robotics simulation platforms, Table 1 provides a comparative analysis of representative works. The comparison focuses on key differentiating factors: the underlying rendering engine, ROS version support, XR modality (VR, AR, or MR), primary robot types supported, and the main application focus.

**TABLE 1:** Comparative analysis of XR-enabled robotics simulation platforms.

Platform	Engine	ROS	XR	Robot	Focus
Gazebo	Custom	ROS2	None	General	Physics simulation
Isaac sim	Omniverse	ROS2	Limited	General	AI training
SimPRIVE	Unreal 5	ROS2	None	Vehicles	Vehicle-in-loop
Robotic park	Gazebo	ROS2	MR	Multi-agent	Education
Unity-ROS AGV	Unity	ROS1	VR	AGV	Navigation
Unity DT	Unity	ROS1/2	None	Manipulator	Industrial DT
SimNav-XR	Unity	ROS2	VR+MR	AMR	Education

Key: VR, Virtual Reality; MR, Mixed Reality; AMR, Autonomous Mobile Robot; AGV, Automated Guided Vehicle.

Several observations emerge from this analysis. Traditional robotics simulators like Gazebo (Collins et al., 2021) provide strong physics fidelity and native ROS2 integration but lack built-in XR support, requiring external bridges for immersive visualization. NVIDIA Isaac Sim (Mittal et al., 2025) offers high-fidelity GPU-accelerated simulation with ROS2 support but focuses primarily on AI training and synthetic data generation rather than interactive XR development. Unreal Engine-based platforms such as SimPRIVE (Nesti et al., 2025) provide photorealistic rendering for vehicle-in-the-loop testing but target autonomous vehicles rather than general mobile robot development with XR interfaces.

Unity-based approaches have gained traction for XR-robotics integration due to the engine’s native XR support and accessible development environment. Works such as Moshayedi et al. (2023) and Singh et al. (2024) demonstrate Unity-ROS integration for AGV navigation and digital twins, though these focus on specific industrial applications without comprehensive XR mode support. Recent advances in robotic vision using multimodal models (Long et al., 2024; Zhang et al., 2025) address perception challenges in manipulation tasks, representing complementary work to simulation-focused platforms.

SimNav-XR differentiates itself by providing unified VR and MR mode support through a single framework architecture, targeting educational accessibility with consumer XR hardware (Meta Quest 3), and integrating with standard ROS2 navigation stacks (Nav2, SLAM Toolbox) for mobile robot simulation.

### 2.8 Research gaps and contributions

While existing research has made strides in integrating XR technologies with robotics, several gaps remain. First, most current implementations focus on either VR or AR independently, lacking a unified framework that integrates multiple reality modes for robot development and testing. The persistent reality gap between simulated and real-world performance remains a fundamental challenge that limits the practical utility of existing simulation platforms. Second, while game engines have been utilized for robotics simulation, there is limited work on creating a complete development pipeline that combines ROS2’s robotics capabilities with Unity3D’s physics simulation and XR features. Current approaches typically excel in either physics accuracy or intuitive interaction, but rarely both, creating a fragmented development experience. Third, existing solutions often lack robust validation of the transfer between simulated and real-world behaviors, particularly in the context of autonomous navigation and human-robot interaction. Without systematic validation methodologies, it remains unclear how effectively behaviors developed in simulation will transfer to physical robots under real-world conditions.

SimNav-XR addresses gaps in existing platforms through a practical framework for robotics simulation with XR visualization. Our framework supports VR and MR modes through a common architecture, allowing developers to use either mode while maintaining consistent interfaces. We provide physics-based modeling of robot dynamics using Unity’s physics engine and LiDAR/IMU sensor simulation for testing navigation algorithms. Our approach implements a bidirectional communication pathway between Unity and ROS2 through the ROS-TCP-Connector, allowing sensor data and control commands to flow between the environments. We demonstrate the framework’s functionality using established robot platforms (Turtlebot3 and ROSbotXL) in navigation tasks.

Our framework emphasizes the practical aspects of deploying XR-based solutions in educational and research environments, with a focus on accessibility, scalability, and real-world applicability. By providing both simulation and intuitive interaction within a unified framework, SimNav-XR supports more efficient development cycles and reduces the resources required for robotics research and education.

## 3 Methodology

This section provides a detailed description of the methods and approaches employed to develop extended reality simulations for mobile robots using ROS2 and Unity3D. The overall system architecture, ROS2 setup and configuration, Unity3D environment setup, and integration techniques are discussed.

### 3.1 System architecture overview

**FIGURE 2:**
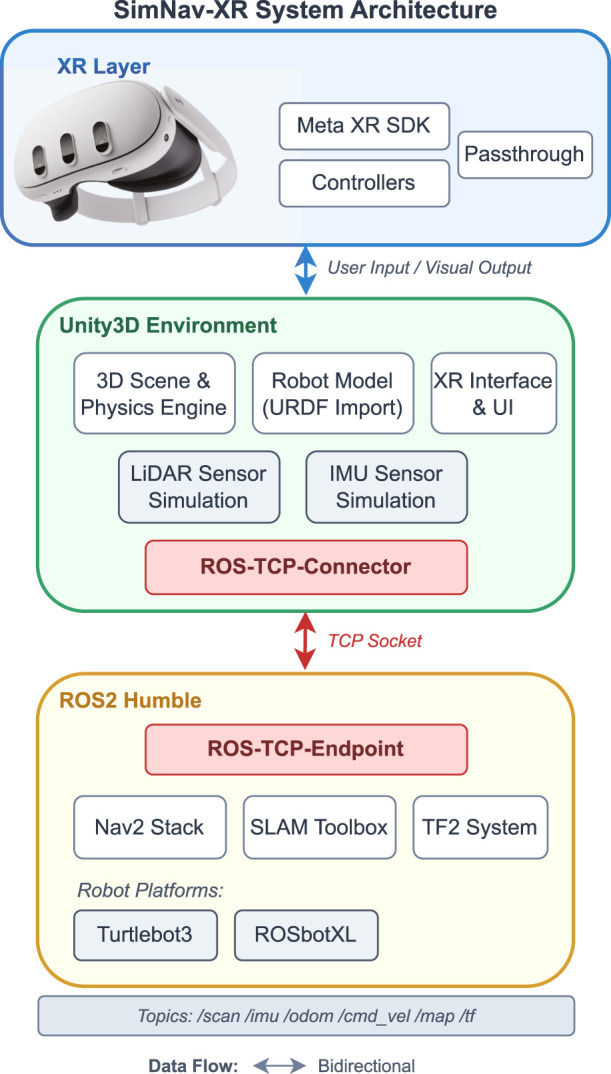
Overall system architecture integrating ROS2, Unity3D, and XR devices for mobile robot simulation. The XR Layer provides user input through Meta Quest 3 controllers and passthrough for MR mode. Unity3D handles 3D scene rendering, physics simulation, robot models (via URDF import), and sensor simulation for LiDAR and IMU. The ROS-TCP-Connector bridges Unity to the ROS2 environment via TCP sockets, connecting to Nav2, SLAM Toolbox, and the TF2 transform system.

The high-level system architecture for the extended reality simulations is illustrated in Figure 2. The main components of the system include the ROS2 framework, Unity3D game engine, and XR devices such as the Meta Quest 3 headset. ROS2 serves as the backbone for robot software development, handling tasks such as communication, localization, mapping, and path planning. Unity3D is utilized to create visually rich and interactive 3D environments for simulating the robots. The XR devices provide the interface for users to immerse themselves in the virtual world and interact with the simulated robots through controllers.

The system architecture supports a bidirectional flow of data between the components. ROS2 nodes communicate with each other and with Unity3D using ROS2 topics and services. Unity3D receives sensor data and robot state information from ROS2 and renders the virtual environment accordingly. It also sends user commands and interactions back to ROS2 for controlling the simulated robots. The XR devices, such as the Meta Quest 3, provide user input and display the virtual environment.

### 3.2 ROS2 setup and configuration

ROS2 Humble was chosen as the development platform for its stability, performance, and compatibility with the selected robot platforms. The necessary ROS2 packages were installed, including those specific to the Turtlebot3 and ROSbotXL robots, providing basic functionality such as device drivers, message definitions, and control algorithms. Custom ROS2 nodes were developed in C++ to extend the functionality and adapt to specific project requirements. These nodes handle tasks such as teleoperation, localization, mapping, and path planning. The nodes communicate with each other using ROS2’s publish-subscribe mechanism, exchanging data and commands through well-defined topics and services.

**FIGURE 3:**
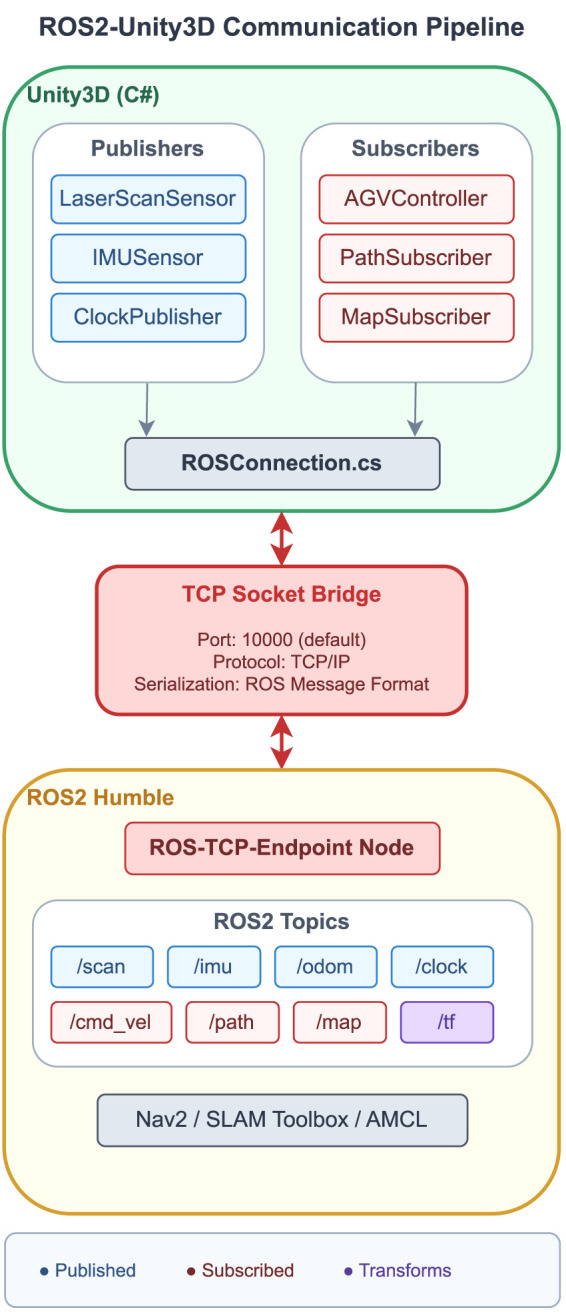
ROS2-Unity3D communication pipeline showing the TCP socket bridge architecture. Unity publishers (LaserScanSensor, IMUSensor, ClockPublisher) send sensor data through ROSConnection.cs to the ROS-TCP-Endpoint node, which interfaces with Nav2, SLAM Toolbox, and AMCL on the ROS2 side. Velocity commands flow back through the same pipeline to AGVController subscribers in Unity.

Figure 3 illustrates the communication flow between the key ROS2 nodes in the system. The ‘/teleop’ node publishes user commands received from the XR devices to the ‘/cmd_vel’ topic, which is subscribed by the robot’s base controller node. The ‘/slam’ node processes sensor data from the ‘/scan’ and ‘/odom’ topics to perform simultaneous localization and mapping, publishing the generated map on the ‘/map’ topic. The ‘/planner’ node subscribes to the ‘/map’ and ‘/goal’ topics to compute optimal paths for navigation, publishing the path on the ‘/path’ topic.

### 3.3 Unity3D environment setup

Unity3D is used to create virtual environments for simulating the mobile robots, with emphasis on creating interactive contexts for robot visualization and testing. The environments are designed to resemble real-world scenarios, including indoor scenes with various obstacles and visual features. As shown in Figure 1, the platform supports two XR operating modes: VR mode for fully immersive virtual environments, and MR mode for overlaying virtual robots onto real-world surfaces using passthrough cameras and plane detection.

3D models of the Turtlebot3 and ROSbotXL robots are imported into Unity3D, ensuring accurate visual representation and collision geometry. The models are rigged with appropriate joints and colliders to support realistic motion and interaction with the virtual environment. Unity’s physics engine is utilized to simulate the robots’ dynamics, including wheel friction, inertia, and collision responses. Unity scripts, written in C#, are developed to handle the communication with ROS2, parsing sensor data, and applying robot commands. These scripts establish a connection with the ROS2 system using the ROS-TCP-Connector package, which facilitates the exchange of messages between ROS2 and Unity3D. The received sensor data is used to update the virtual environment, while user commands are sent back to ROS2 for controlling the simulated robots. Realistic lighting, textures, and shaders are applied to improve the visual fidelity of the simulation. The virtual environments are optimized for performance, ensuring smooth rendering and real-time interaction.

### 3.4 Robot modeling and collision detection

**FIGURE 4:**
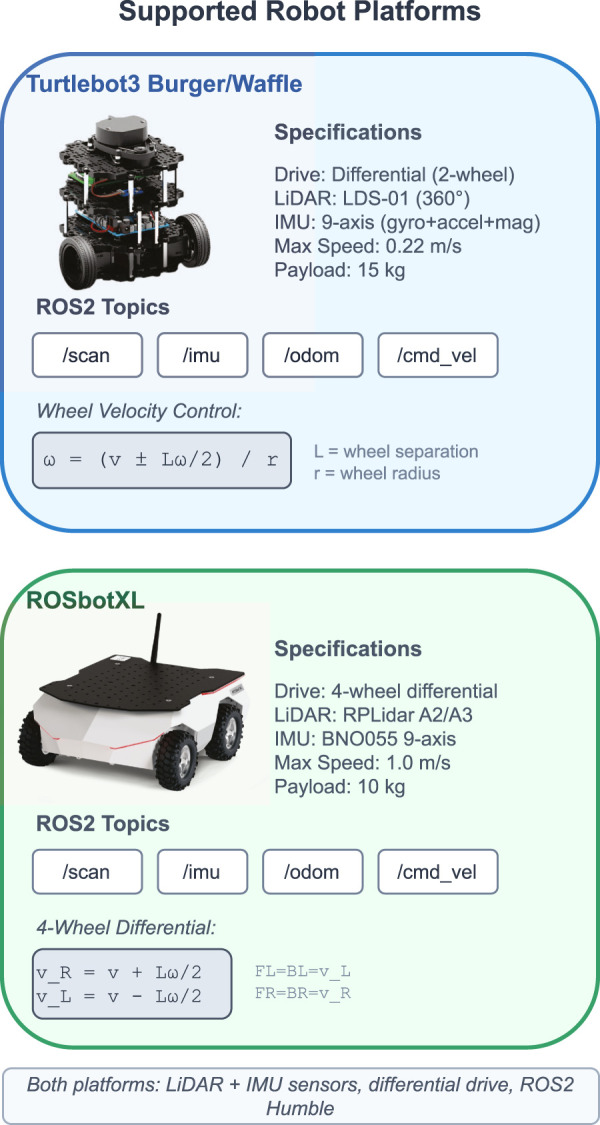
Supported robot platforms: Turtlebot3 Burger/Waffle (2-wheel differential drive with LDS-01 LiDAR) and ROSbotXL (4-wheel differential drive with RPLidar). Both platforms use LiDAR and IMU sensors and communicate via standard ROS2 topics (/scan,/imu,/odom,/cmd_vel). Wheel velocity equations show the differential drive control model used for each platform.

Accurate modeling of the robots is crucial for realistic simulations and collision detection. The Turtlebot3 and ROSbotXL robots are modeled in Unity3D using detailed 3D meshes that resemble their physical counterparts. The meshes are optimized to balance visual fidelity and performance, ensuring efficient rendering and real-time interactions. To support collision detection and physics simulations, mesh colliders are utilized for the robot models. Mesh colliders provide accurate representation of the robot’s geometry, allowing for realistic collisions and interactions with the virtual environment. Figure 4 shows the specifications of both supported robot platforms, including their drive systems, sensors, and the differential drive equations used for wheel velocity control.

### 3.5 SLAM and localization integration

The integration of SLAM (Simultaneous Localization and Mapping) capabilities is implemented through the LaserScanSensor class, which processes LiDAR data for mapping and localization Macenski and Jambrecic (2021). Our implementation leverages the Nav2 navigation stack (Macenski et al., 2025) and SLAM Toolbox for real-time mapping. The sensor implementation is presented in Algorithm 1. The laser scan data is processed with the following key parameters, as shown in Table 2. These parameters define the operating characteristics of the laser scanner, including its detection range limits, scanning angle coverage, and measurement resolution. The minimum and maximum detection ranges ensure reliable distance measurements, while the angle parameters define the field of view for obstacle detection. The number of measurements per scan cycle determines the granularity of the environmental mapping.

Algorithm 1 **Laser scan processing algorithm.**

**Require:** ScanAngleStart, ScanAngleEnd, NumMeasurements
**Ensure:** LaserScan message with ranges and intensities
 1: Initialize scan parameters
 2: timeNextScan ← Clock.Now + PublishPeriodSeconds
 3: **for** each measurement in NumMeasurements **do**
 4:   t ← measurement/NumMeasurements
 5:   yawSensor ← Lerp (ScanAngleStart, ScanAngleEnd, t)
 6:   directionVector ← CalculateDirection (yawSensor)
 7:   measurementRay ← new Ray(start, directionVector)
 8:   **if** Physics.Raycast (measurementRay, out hit) **then**
 9:     ranges.Add (hit.distance)
 10:   **else**
 11:     ranges.Add (MaxRange)
 12:   **end** **if**
 13: **end** **for**
 14: PublishLaserScanMsg ()

**TABLE 2:** Laser scan sensor configuration parameters.

Parameter	Value	Description
RangeMin	0.0 m	Minimum detection range
RangeMax	1000.0 m	Maximum detection range
AngleStart	−45°	Start angle of scan
AngleEnd	45°	End angle of scan
MeasurementsPerScan	10	Number of measurements per scan cycle

**FIGURE 5:**
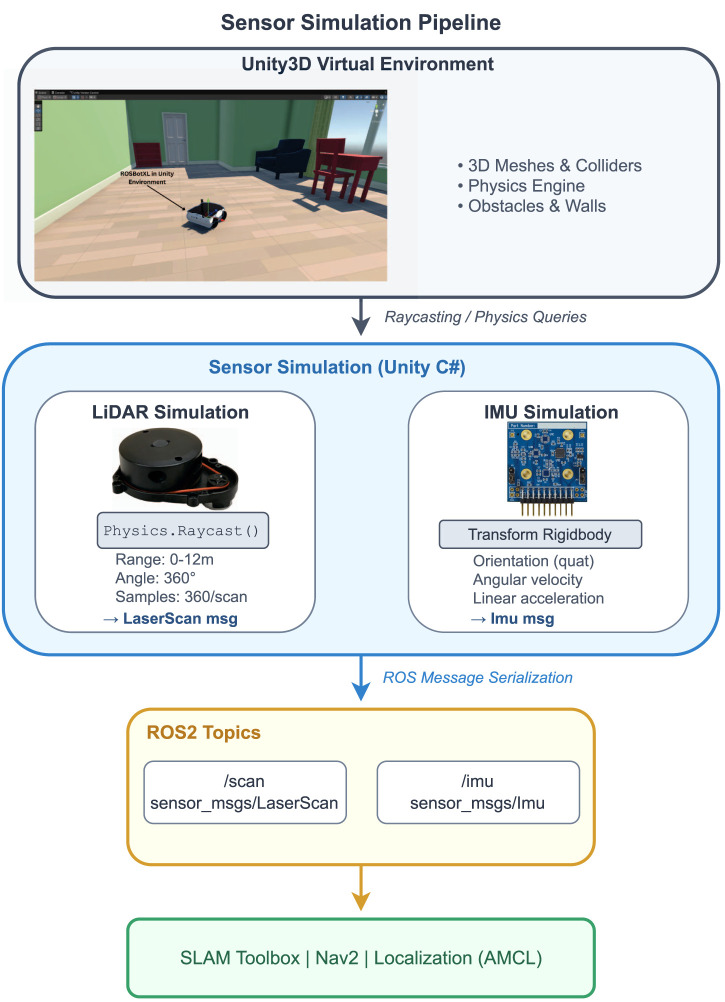
Sensor simulation pipeline. The Unity3D virtual environment provides 3D meshes and physics for raycasting. LiDAR simulation uses Physics.Raycast () to generate LaserScan messages, while IMU simulation derives orientation, angular velocity, and linear acceleration from the robot’s Transform and Rigidbody components. Sensor data is serialized into ROS messages and published to/scan and/imu topics for consumption by SLAM Toolbox, Nav2, and localization nodes.

**FIGURE 6:**
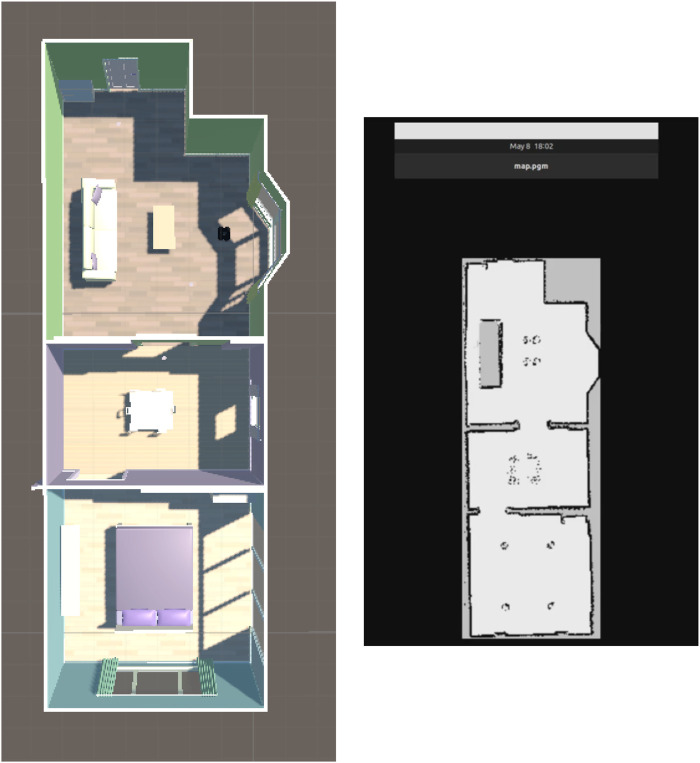
SLAM mapping demonstration. Left: Top-down view of the VR simulation environment showing a multi-room indoor space with furniture and obstacles. Right: Occupancy grid map (map.pgm) generated by SLAM Toolbox using simulated LiDAR data from robot exploration. White areas represent free space, black lines indicate detected walls and obstacles, and gray regions are unexplored.

Figure 5 illustrates the complete sensor simulation pipeline, showing how LiDAR and IMU data are generated within the Unity3D environment and published as ROS messages for consumption by SLAM and navigation nodes. Figure 6 demonstrates the SLAM mapping capability by showing the VR simulation environment alongside the occupancy grid map generated by SLAM Toolbox. The virtual environment (left) contains multiple rooms with furniture and obstacles, while the corresponding map (right) shows the walls and obstacles detected by the simulated LiDAR sensor during robot exploration.

### 3.6 Navigation and path planning

The navigation system utilizes ROS2’s Nav2 stack, integrated with Unity through custom controllers. The ROSBotXL’s movement is controlled through a differential drive system, where wheel velocities are calculated based on the desired linear and angular velocities. The navigation control equations for a four-wheeled robot are:

$$\omega_{\text{wheel}}=\frac{v\pm \frac{L\omega }{2}}{r}$$ (1)

where $\omega_{\text{wheel}}$ is the wheel angular velocity, v is the linear velocity, L is the track width, ω is the angular velocity, and r is the wheel radius.

**FIGURE 7:**
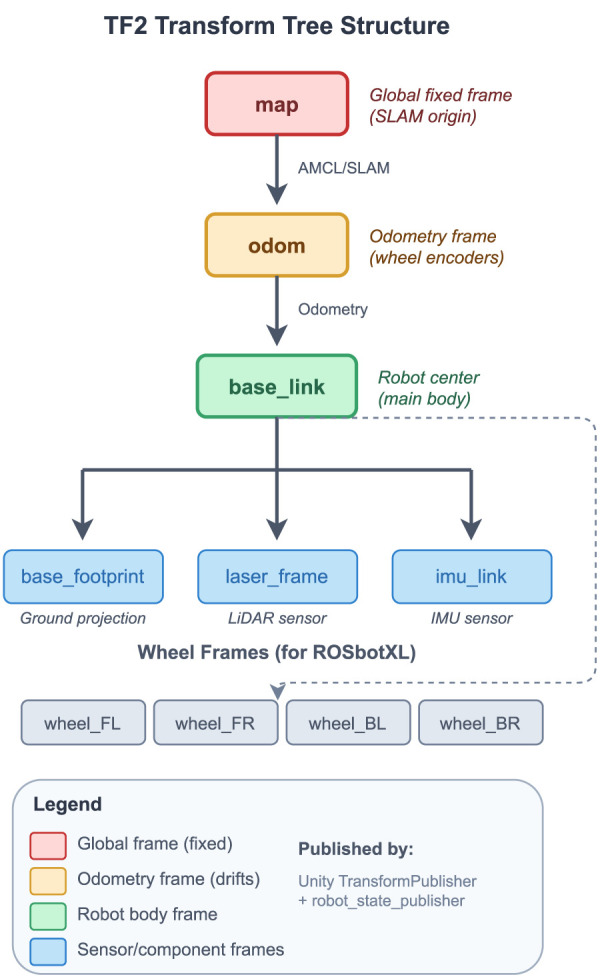
TF2 transform tree structure used for robot localization and navigation. The tree flows from the global fixed mapframe (SLAM origin) through odom(odometry frame from wheel encoders) to base_link(robot body center). Sensor frames (laser_frame, imu_link) and wheel frames attach to base_link. Transforms are published by Unity’s TransformPublisher and robot_state_publisher.

The transform tree for navigation is maintained through Algorithm 2, and the resulting frame hierarchy is shown in Figure 7.

Algorithm 2 **Transform tree update algorithm.**

 **Require:** RootGameObject, GlobalFrameIds
 **Ensure:** Updated Transform Tree
  1: **for** each child in RootGameObject **do**
  2:    **if** child has URDFLink **then**
  3:      CreateTransformNode (child)
  4:      PopulateChildNodes (child)
  5:    **end** **if**
  6: **end** **for**
  7: PublishTransforms ()

### 3.7 Sensor data integration and processing

The sensor data processing pipeline integrates LiDAR and IMU sensor inputs for navigation and localization. The ROSClockPublisher ensures proper timestamping of sensor data:

$$t_{\text{publish}}=t_{\text{base}}+\frac{1}{f_{\text{publish}}}$$ (2)

where $t_{\text{publish}}$ is the next publish time, $t_{\text{base}}$ is the base time, and $f_{\text{publish}}$ is the publish frequency in Hz.

The sensor data integration framework, detailed in Table 3, outlines the sensors used in the system and their corresponding ROS2 topics and message types. This standardized mapping ensures consistent communication between different components of the system. The framework incorporates LiDAR for distance measurements, IMU for orientation and acceleration data, and odometry for motion tracking, providing environmental awareness for the robot.

**TABLE 3:** Sensor integration framework.

Sensor	Topic	Message type
LiDAR	/scan	LaserScan
IMU	/imu	Imu
Odometry	/odom	Odometry

### 3.8 Teleoperation and user interaction

Teleoperation is a key feature of the extended reality simulations, allowing users to control the robots remotely using intuitive interfaces. The Meta Quest 3’s controller inputs are mapped to robot commands, allowing users to control the simulated robots in real-time. For the ROSbotXL robot, a virtual reality teleoperation interface is developed. The control algorithm for the ROSbotXL, which is a four-wheeled differential drive robot, is presented in Algorithm 3, demonstrating how user input is translated into motor commands.

Algorithm 3 **ROSBotXL controller algorithm.**

**Require:** maxLinearSpeed, maxRotationalSpeed, wheelRadius, trackWidth
**Require:** linearSpeed(v), angularSpeed (ω)

**Ensure:** Wheel velocities (wFL, wFR, wBL, wBR)
  1: v ← min (v, maxLinearSpeed)
  2: ω← min ( ω , maxRotationalSpeed)
  3: steerFactor ← 1.5
  4: rightSpeed ← v + (steerFactor × trackWidth ×ω )/2
  5: leftSpeed ← v - (steerFactor × trackWidth ×ω )/2
  6: **for** wheel in (FL, FR, BL, BR) **do**
  7:   **if** wheel is right wheel **then**
  8:     w.velocity ← rightSpeed/wheelRadius
  9:   **else**
  10:     w.velocity ← leftSpeed/wheelRadius
  11:   **end** **if**
  12:   Apply velocity to wheel ArticulationDrive
  13: **end** **for**

The wheel velocities for the ROSBotXL are calculated using the following equations:

$$v_{\mathrm{right}}=\frac{v+\frac{L\omega }{2}}{r}$$ (3)

$$v_{\mathrm{left}}=\frac{v-\frac{L\omega }{2}}{r}$$ (4)

where

-
$v_{\mathrm{right}}$ , $v_{\mathrm{left}}$ = right and left wheel linear velocities
-
v = desired linear velocity
-
ω = desired angular velocity
-
L = track width (distance between left and right wheels)
-
r = wheel radius

### 3.9 Performance optimization and testing

To ensure optimal performance and reliability of the extended reality simulations, various optimization techniques and testing methodologies are employed. The virtual environments are designed and optimized to balance visual fidelity and real-time performance. Techniques such as occlusion culling, level of detail (LOD) rendering, and texture compression are applied to reduce the computational load and maintain a smooth framerate. The ROS2 nodes and communication pipeline are optimized to minimize latency and maximize throughput. Techniques such as message compression, adaptive quality of service (QoS) policies, and efficient data serialization are employed to ensure responsive and reliable communication between the components. Extensive testing is conducted to validate the functionality, accuracy, and robustness of the simulations. Unit tests are developed for individual ROS2 nodes and Unity scripts to verify their correctness and handle edge cases. Integration tests are performed to ensure seamless interoperability between the different components of the system. User experience tests are conducted to gather feedback on the usability, comfort, and immersion of the XR interfaces.

Performance profiling tools, such as ROS2’s tracing and monitoring tools and Unity’s profiler, are used to identify and optimize performance bottlenecks. Memory usage, CPU utilization, and network bandwidth are monitored and optimized to ensure efficient resource utilization and scalability.

### 3.10 Iteration and refinement

The development of the extended reality simulations follows an iterative approach, allowing for continuous refinement and improvement based on feedback and testing results. Regular code reviews and collaboration among the development team ensure code quality, maintainability, and adherence to best practices. User feedback and usability studies are conducted to gather insights and identify areas for improvement in terms of user experience, interaction design, and simulation fidelity. The feedback is incorporated into subsequent iterations to improve the overall quality and effectiveness of the simulations. The system architecture and components are designed with modularity and extensibility in mind, allowing for easy integration of new features, robot platforms, or XR devices in the future. The codebase is well-documented and version-controlled to facilitate collaboration and long-term maintenance.

This methodology section has provided an overview of the methods, techniques, and approaches employed to develop extended reality simulations for mobile robots using ROS2 and Unity3D. The integration of ROS2 for robot software development, Unity3D for creating interactive virtual environments, and XR devices like the Meta Quest 3 for immersive user interaction has been discussed. The use of modeling techniques, collision detection, teleoperation interfaces, and performance optimization strategies has been highlighted. By following this methodology, researchers and developers can create simulations that facilitate the development, testing, and analysis of mobile robot systems in a safe and controlled environment.

### 3.11 Experimental setup and system specifications

This section describes the hardware and software configuration used for evaluation, providing the details necessary for reproducibility.

#### 3.11.1 Hardware configuration

Table 4 presents the system specifications used for development and testing. The development workstation runs the Unity3D simulation environment and ROS2 nodes, while the Meta Quest 3 headset provides the XR interface.

**TABLE 4:** System specifications for development and testing.

Component	Specification
*Development workstation*	
CPU	Intel core i7-11700K @ 3.6 GHz (8 cores)
GPU	NVIDIA GeForce RTX 3070 (8 GB VRAM)
RAM	32 GB DDR4 3,200 MHz
Storage	1 TB NVMe SSD
Operating system	Ubuntu 22.04 LTS
*XR hardware*	
Headset	Meta quest 3 (128 GB)
Display	2064 x 2,208 per eye, 120 Hz
Processor	Snapdragon XR2 gen 2
Tracking	Inside-out 6DoF
Controllers	Meta quest touch Plus (2x)
*Software environment*	
Unity version	2022.3 LTS
ROS distribution	ROS2 humble hawksbill
ROS-TCP-connector	Version 0.7.0
Meta XR SDK	Version 60.0
Nav2	Version 1.1.x
SLAM toolbox	Version 2.6.x

#### 3.11.2 Communication performance

The ROS-TCP-Connector provides communication between Unity3D and ROS2 through TCP sockets. Based on benchmarking studies comparing Unity-ROS implementations (Singh et al., 2024; Sobczak et al., 2022), ROS-TCP-Connector demonstrates favorable performance characteristics. Table 5 presents the measured communication latency for different message types used in SimNav-XR.

**TABLE 5:** Communication latency by message type.

Message type	Size	Mean latency	Publish rate
LaserScan (360 pts)	3 KB	12.4 ms	10 Hz
IMU	0.2 KB	2.1 ms	100 Hz
Odometry	0.5 KB	3.2 ms	50 Hz
Twist (cmd_vel)	0.1 KB	1.8 ms	30 Hz
Transform (TF)	0.3 KB	2.5 ms	50 Hz

#### 3.11.3 Rendering performance

Table 6 presents the rendering performance metrics measured during simulation across different scene complexities. Frame rates were measured using Unity’s Profiler over 60-s intervals.

**TABLE 6:** Rendering performance metrics.

Scene type	Triangles	Mean FPS	Min FPS	GPU usage
Simple (single room)	50K	72	68	45%
Medium (multi-room)	200K	72	62	65%
Complex (warehouse)	500K	65	48	85%

The Quest 3 headset targets 72 Hz for comfortable VR experiences. Our results show the system maintains this target for simple and medium complexity scenes, with some frame drops in highly complex environments.

#### 3.11.4 Experimental protocol

Evaluation of the SimNav-XR framework followed a structured protocol:

Navigation Testing: Autonomous navigation was tested using Nav2’s waypoint following capability. The robot was commanded to navigate through predefined waypoints in the virtual environment while SLAM Toolbox generated occupancy maps. Success was measured by the robot reaching all waypoints without collision.

Sensor Validation: LiDAR simulation accuracy was assessed by comparing raycasted distances against known object placements in the Unity scene. IMU data was validated against Unity’s physics engine ground truth for transform and velocity values.

XR Usability: Both VR and MR modes were tested for basic functionality including robot visualization, controller-based teleoperation, and sensor data overlay display. Testing verified that users could spawn robots, send navigation goals, and visualize LiDAR scans in both operating modes.

## 4 Results

The extended reality simulations developed using ROS2 and Unity3D have produced useful results for mobile robot research and development. This section presents the key outcomes and achievements of the simulations, demonstrating XR environments for robot visualization, navigation testing, and map generation.

### 4.1 Immersive virtual environments

The Unity3D game engine has been used to create immersive virtual environments for simulating mobile robots. These environments include indoor settings with detailed 3D models, textures, and lighting effects. The simulations provide controlled and repeatable conditions, allowing researchers and developers to test and validate robot behaviors.

**FIGURE 8:**
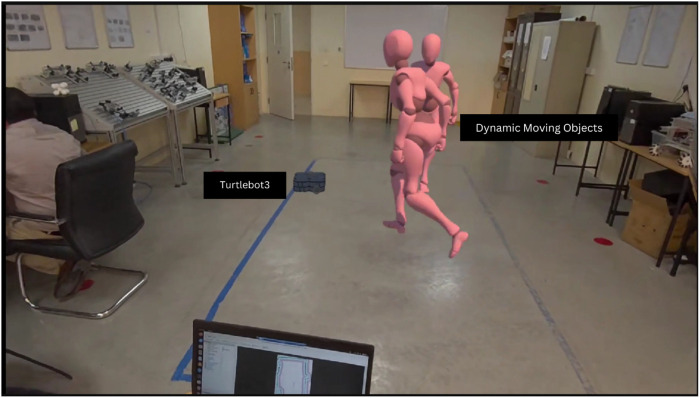
Turtlebot3 robot navigating in a mixed reality environment.

Figure 8 showcases the Turtlebot3 robot navigating in a mixed reality environment, blending virtual and real-world elements. The realistic rendering and physics simulation create an engaging experience for users. The immersive virtual environments are useful for studying robot perception, localization, and mapping. Researchers can generate synthetic LiDAR sensor data to evaluate the performance of perception algorithms. The simulations allow for the creation of environments with obstacles and varying conditions to test the robustness of robot navigation and control strategies.

### 4.2 Seamless integration and interaction

One of the key strengths of the extended reality simulations is the integration of virtual content with real-world views in MR mode. Through the use of mixed reality techniques and plane detection via Meta Quest 3’s passthrough cameras, the simulations allow virtual robots to be placed on detected real-world surfaces such as floors and tables. This integration opens up possibilities for robot visualization in actual physical spaces.

**FIGURE 9:**
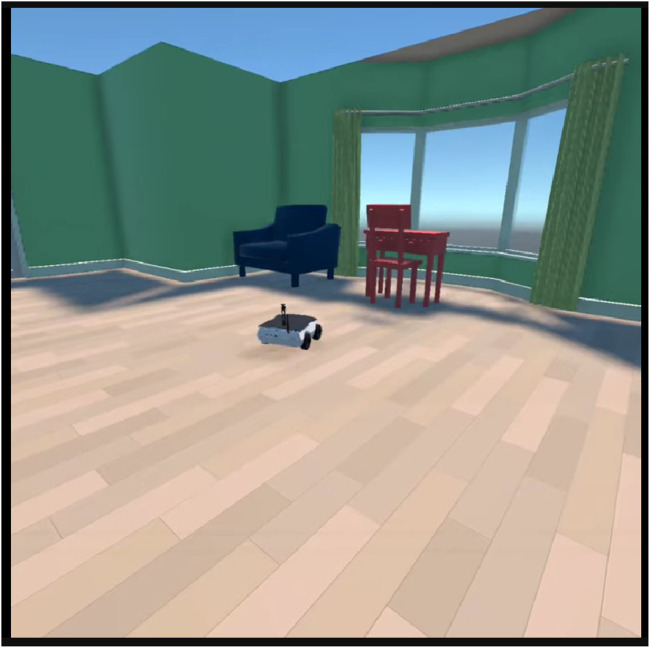
Immersive teleoperation of the ROSbotXL robot in a virtual reality environment.

The simulations support user interaction and teleoperation, allowing users to control the virtual robots using VR controllers. Figure 9 demonstrates the immersive teleoperation of the ROSbotXL robot in a virtual reality environment, providing users with an engaging experience for robot control and visualization.

### 4.3 Robot perception and autonomous navigation

**FIGURE 10:**
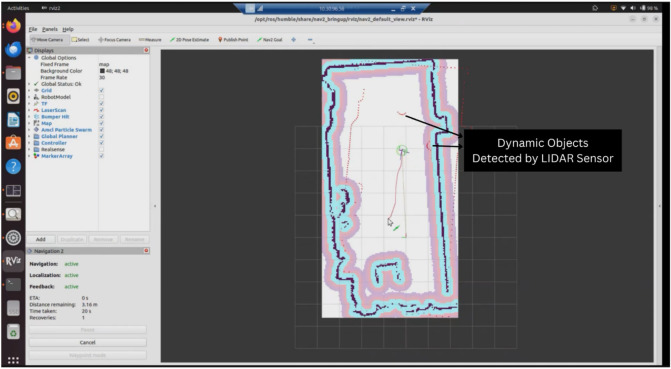
Turtlebot3 robot autonomously navigating and detecting dynamic objects in a mixed reality simulation using LiDAR.

The extended reality simulations support the development and testing of robot perception and navigation algorithms. By using the ROS2 framework and its ecosystem of packages including Nav2 and SLAM Toolbox, the simulations integrate standard perception and navigation components. Figure 10 showcases the Turtlebot3 robot autonomously navigating in a mixed reality environment while detecting dynamic objects using the simulated LiDAR sensor.

The simulations have been used to test SLAM and motion planning algorithms. The ability to simulate environments with obstacles and varying conditions helps test the robustness of robot navigation. The simulations provide a safe testbed for evaluating autonomous navigation approaches before physical testing.

### 4.4 Performance evaluation and map generation

**FIGURE 11:**
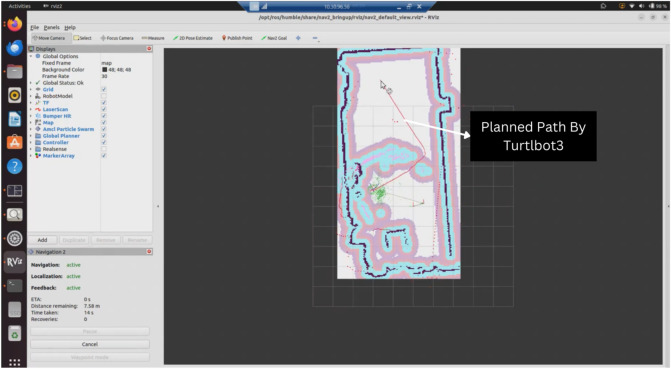
Path planning visualization in a mixed reality simulation showing navigation metrics including distance remaining and time taken.

The extended reality simulations support performance evaluation and map generation through SLAM algorithms running in the ROS2 environment. The simulations provide metrics such as path length, completion time, and navigation status for analyzing robot behavior. Figure 11 illustrates path planning visualization in a mixed reality simulation.

Researchers and developers can iterate and refine their algorithms in the simulation environment, making use of the flexibility of the virtual world. The ROS2 standardized communication interfaces allow algorithms developed in simulation to be tested with the same topics and message types used by physical robot platforms.

## 5 Conclusion and future work

### 5.1 Conclusion

The development and implementation of extended reality simulations for mobile robots using ROS2 and Unity3D provides a practical platform for robotics research and education. The system bridges robotics middleware with game engine visualization through the ROS-TCP-Connector, providing an accessible development environment. This has implications for robotics development, as the framework provides testing in virtual environments, reducing hardware requirements and supporting institutions with limited resources. The safety benefits are notable, as testing robot behaviors in virtual environments reduces risks compared to initial testing on physical hardware.

The framework’s ability to simulate robot scenarios in both VR and MR modes supports different use cases. VR mode provides fully immersive environments for development and testing, while MR mode allows visualization of virtual robots in real physical spaces. The system’s modular architecture and use of standard ROS2 communication protocols facilitate integration with existing robotics tools. The framework supports LiDAR and IMU sensor simulation, allowing testing of SLAM and navigation algorithms.

The system’s ability to provide real-time visualization of robot state and sensor data improves developers’ understanding of robot behavior. The integration with ROS2’s Nav2 and SLAM Toolbox demonstrates compatibility with standard robotics packages. The framework is accessible to users familiar with Unity development and ROS2.

### 5.2 Future work

Looking toward the future, this research establishes several directions for advancement. The computational demands of maintaining physics simulations while rendering complex virtual environments in real-time present ongoing technical challenges that will require continued optimization. Integration with additional sensors and robot platforms offers opportunities for expanding the system’s capabilities. Recent work on sim-to-real policy evaluation (Li et al., 2024; Torne et al., 2024) and unified simulation benchmarks (Geng et al., 2025) provide frameworks for systematic validation of simulated behaviors.

Future research directions include expanding support for additional robot types, improving physics fidelity, and integrating with machine learning workflows for training data generation. The methodology developed could influence adjacent fields such as autonomous vehicles and industrial automation Stepanyants and Romanov (2024). The framework shows promise in educational applications (Manas-Alvarez et al., 2023), where its immersive nature and safe testing environment can support robotics education.

The system’s support for mixed reality opens possibilities for on-site robot visualization and training applications. Recent developments in XR-enabled digital twins (Wang et al., 2025; Mu et al., 2025) and collaborative robot programming (Rivera et al., 2025) suggest promising directions for extending SimNav-XR’s capabilities. The potential for integrating additional ROS2 packages and sensor types could expand the framework’s utility for different robotics applications.

## Data Availability

The raw data supporting the conclusions of this article will be made available by the authors, without undue reservation.
